# Evaluation of the effects of cell-dispensing using an inkjet-based bioprinter on cell integrity by RNA-seq analysis

**DOI:** 10.1038/s41598-020-64193-z

**Published:** 2020-04-28

**Authors:** Masayuki Yumoto, Natsuko Hemmi, Naoki Sato, Yudai Kawashima, Koji Arikawa, Keigo Ide, Masahito Hosokawa, Manabu Seo, Haruko Takeyama

**Affiliations:** 1 Department of Life Science and Medical Bioscience, Waseda University, 2-2 Wakamatsu-cho, Shinjuku-ku, Tokyo, 162-8480 Japan; 2 Biomedical Business Center, Healthcare Business Group, Ricoh Company, Ltd., 3-25-22 Tonomachi LIC 322, Kawasaki, Kanagawa 210-0821 Japan; 3 Computational Bio Big-Data Open Innovation Laboratory, AIST-Waseda University, 3-4-1 Okubo, Shinjuku-ku, Tokyo, 169-8555 Japan; 4 Institute for Advanced Research of Biosystem Dynamics, Waseda Research Institute for Science and Engineering, Waseda University, 3-4-1 Okubo, Shinjuku-ku, Tokyo, 169-8555 Japan; 5 Research Organization for Nano and Life Innovation, Waseda University, 513 Waseda-tsurumaki-cho, Shinjuku-ku, Tokyo, 162-0041 Japan

**Keywords:** Bioinformatics, RNA sequencing, Tissue engineering, Transcriptomics

## Abstract

Bioprinting technology is expected to be applied in the fields of regenerative medicine and drug discovery. There are several types of bioprinters, especially inkjet-based bioprinter, which can be used not only as a printer for arranging cells but also as a precision cell-dispensing device with controlled cell numbers similar to a fluorescence activated cell sorter (FACS). Precise cell dispensers are expected to be useful in the fields of drug discovery and single-cell analysis. However, there are enduring concerns about the impacts of cell dispensers on cell integrity, particularly on sensitive cells, such as stem cells. In response to the concerns stated above, we developed a stress-free and media-direct-dispensing inkjet bioprinter. In the present study, in addition to conventional viability assessments, we evaluated the gene expression using RNA-seq to investigate whether the developed bioprinter influenced cell integrity in mouse embryonic stem cells. We evaluated the developed bioprinter based on three dispensing methods: manual operation using a micropipette, FACS and the developed inkjet bioprinter. According to the results, the developed inkjet bioprinter exhibited cell-friendly dispensing performance, which was similar to the manual dispensing operation, based not only on cell viability but also on gene expression levels.

## Introduction

Bioprinting technology has grown in tandem with stem cell research, and it has become a vital tool with diverse applications in the biological and medical fields. Artificial tissues or organs fabricated using bioprinters are expected to be used in regenerative medicine and drug discovery^1,2^. There are several types of bioprinters, and inkjet-based bioprinters possess a specification to print a single cell^3^ or cell aggregates^4^ by controlling process parameters, such as nozzle diameter, droplet volume and cell concentration^5^. Owing to the specification mentioned above, inkjet-based bioprinter can be used not only as a printer for arranging cells but also as a precision cell-dispensing device with controlled cell numbers.

Precise cell-dispensing technology is expected to be particularly useful in the fields of drug discovery and single-cell analysis. In the field of drug discovery, high-throughput screening of candidate drugs using cell microarrays^6^ is expected to contribute to the reduction of costs for the development of new drugs. Cell microarrays containing some types of cells, especially disease-specific induced pluripotent stem cells (iPSCs) with controlled cell numbers on a chip, are expected to be applied to screen drug efficacy evaluation, toxicity testing and RNAi^7,8^ In addition, cell arrays containing individually-derived iPSCs are expected to be used as personalised pharmaceutics tools^2^. In the field of single-cell analysis, technology for precise and high-throughput dispensing of single-cell is required with remarkable progress of single-cell genome sequencing and single-cell RNA-seq^9–11^. FACS is a mainstream tool in the field of precision cell dispensing with controlled cell numbers in general, but inkjet-based bioprinter can also be used for such applications. FACS machine fundamentally employs the droplet manipulation technology of continuous inkjet system^12,13^. Inkjet bioprinter is categorized into the drop-on-demand system. In this point of view, inkjet bioprinter and FACS are based on the same technique which encapsulate cells in the droplet ejected from the nozzle. Although FACS being the gold standard for high-speed precision cell-dispensing equipment, there is concern about the damage that FACS can cause while dispensing sensitive cells. In particular, there is concern about the effects of chemical stresses from the sheath fluid^14^, mechanical stresses, such as shear stress, as cells pass through the long sample line, and pressure and inertia forces that are exerted when the droplets lands^15^. This concern also exists for the inkjet bioprinters reported so far^5,16,17^. Inkjet bioprinters are categorized into two types: piezoelectric or thermal inkjet type printer. In addition to mechanical stress which are concerns about piezoelectric inkjet bioprinter, thermal inkjet bioprinters are also concerned about heat shock during the printing process^18–20^.

In response to the concerns above, we have developed an inkjet-based bioprinter with a structure that is as stress-free as possible^21^. Our inkjet bioprinter based on piezoelectric inkjet head. This inkjet head has minimal shear stress because of the absence of long narrow channels using disk membrane nozzle with a bending-type piezoelectric actuator, and the internal pressure does not increase because of the presence of an open chamber. In addition, by setting the ejection conditions of the inkjet head to a droplet diameter of ~100 μm and a flying speed of <1 m/s, the pressure and inertial force upon landing is suppressed. In the preparation of bioink for ejecting cells with the present inkjet head, no additional material such as hydrogel is required; therefore, there is no chemical stress on the cells. In this report, to investigate whether the developed inkjet bioprinter is cell-friendly, we performed RNA-seq on cells dispensed using the developed inkjet head and conducted gene expression analyses, in addition to the conventional assessment of cell viability, proliferation and stem cell pluripotency. After dispensing bioink containing mouse embryonic stem cells (mESCs) into a container using the inkjet bioprinter and culturing them for a predetermined period, cell integrity was evaluated. For comparison with the developed inkjet printer, we used a manual method using a micropipette and a fluorescence activated cell sorter (FACS), the gold standard droplet-based precision live-cell dispenser. It is important to know if there are any potential effects that would not be apparent to life or death in order to apply bioprinters in the various applications of cell dispensing. Our results demonstrate that the developed inkjet bioprinter has cell-friendly dispensing capabilities comparable to manual pipetting, not only in terms of cell viability but also with regard to the gene expression variation at transcriptome levels.

## Results and Discussion

### Dispensing conditions of the developed inkjet head and experimental workflow

**Figure 1:**
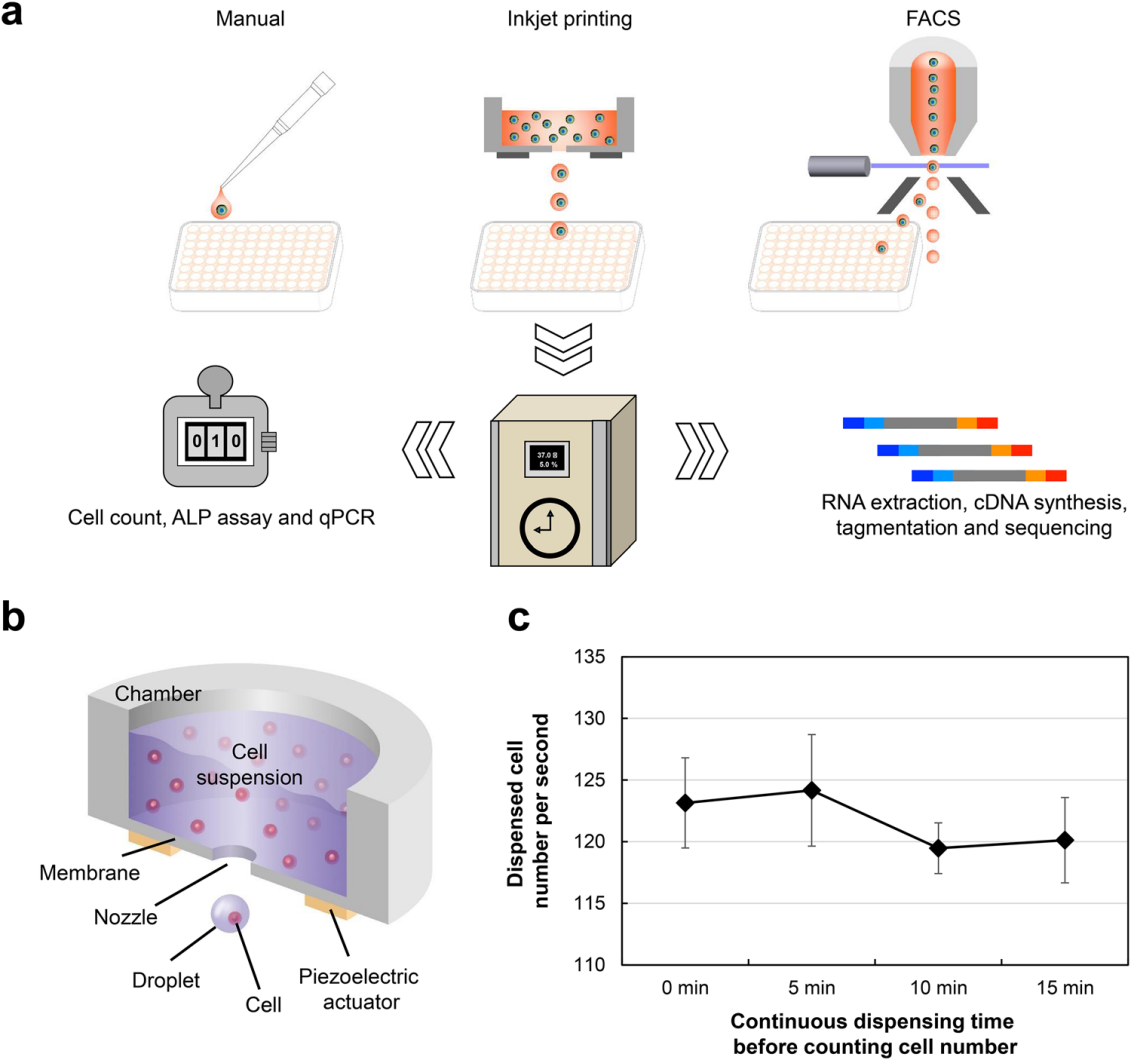
Developed inkjet head and experimental overview. (**a**) Schematic diagram of experimental workflow. mESCs were dispensed into well plates containing culture medium by three methods. After culturing for the appropriate time, the viability, proliferation, maintenance of undifferentiated state and differentiation ability to three germ layers were evaluated using mESCs. Furthermore, total RNA was extracted to conduct RNA-seq. (**b**) Schematic cross-sectional three-dimensional (3D) view of the developed inkjet head. The cell suspension was injected into the chamber. When the membrane was vibrated using the piezoelectric actuator, droplets holding cells were generated from the nozzle. (**c**) Confirmation of ejection stability of the developed inkjet head. Results are presented as mean ± SD of the eighths replicate data.

We evaluated the effect of the developed inkjet head on cells using the workflow as shown in Fig. 1a. We developed the inkjet head and inkjet-based bioprinter in house^21^. This inkjet head comprised an open-air chamber for holding the cell suspension, a membrane fixed to the bottom of the chamber, a nozzle with an opening at the centre of the membrane and an annular piezoelectric actuator fixed outside and below the membrane (Fig. 1b). Cell dispensing using the developed inkjet bioprinter easily corresponded to various platforms ranging from 15-mL tubes to 384-well plates, and it was possible to precisely arrange the cells. In addition, a previous study presented cases where cells were arranged at a 500-μm pitch^21^. Such dispensing accuracy is still observed even when using 1536-well plates, and the inkjet bioprinter supports the specifications of a cell-dispensing device.

In this experiment, we used the inkjet head with a nozzle of 100-μm diameter at the centre of a vibration membrane of 50-μm thickness. The 100-μm diameter nozzle was selected because this diameter is sufficiently larger than that of the cells to reduce shear stress when the cells pass through the nozzle and is the same diameter as the nozzle used in FACS when dispensing stem cells. Before the assessment of cell integrity, we confirmed the cell-dispensing condition of the developed inkjet head. When the 2.0 × 10^6^ cells/ml mESCs suspension was injected into the room temperature chamber, the droplets that included the mESCs (approximately 606 pl volume with 105-μm diameter at a flying speed of 0.6 m/sec) were generated by membrane vibration. If the mESCs suspension is dispensed at 100 Hz of droplet frequency under these conditions, it is calculated that 121 cells are dispensed per second. Further, we continued to dispense the cells and counted the number of dispensed cells per second at the initial stage and after 5 min, 10 min and 15 min (Fig. 1c). The average numbers of dispensed cells per second at a 100-Hz dispensing rate were 123, 124, 119 and 120 cells at the initial stage and after 5 min, 10 min and 15 min of continuous dispensing, respectively. From these results, we confirmed that the developed inkjet head could continuously dispense a stable number of mESCs without causing nozzle clogging, which is a problem in conventional inkjet bioprinters.

### Conventional evaluations of the dispensed mESCs: Cell viability, proliferation and colony formation

**Figure 2:**
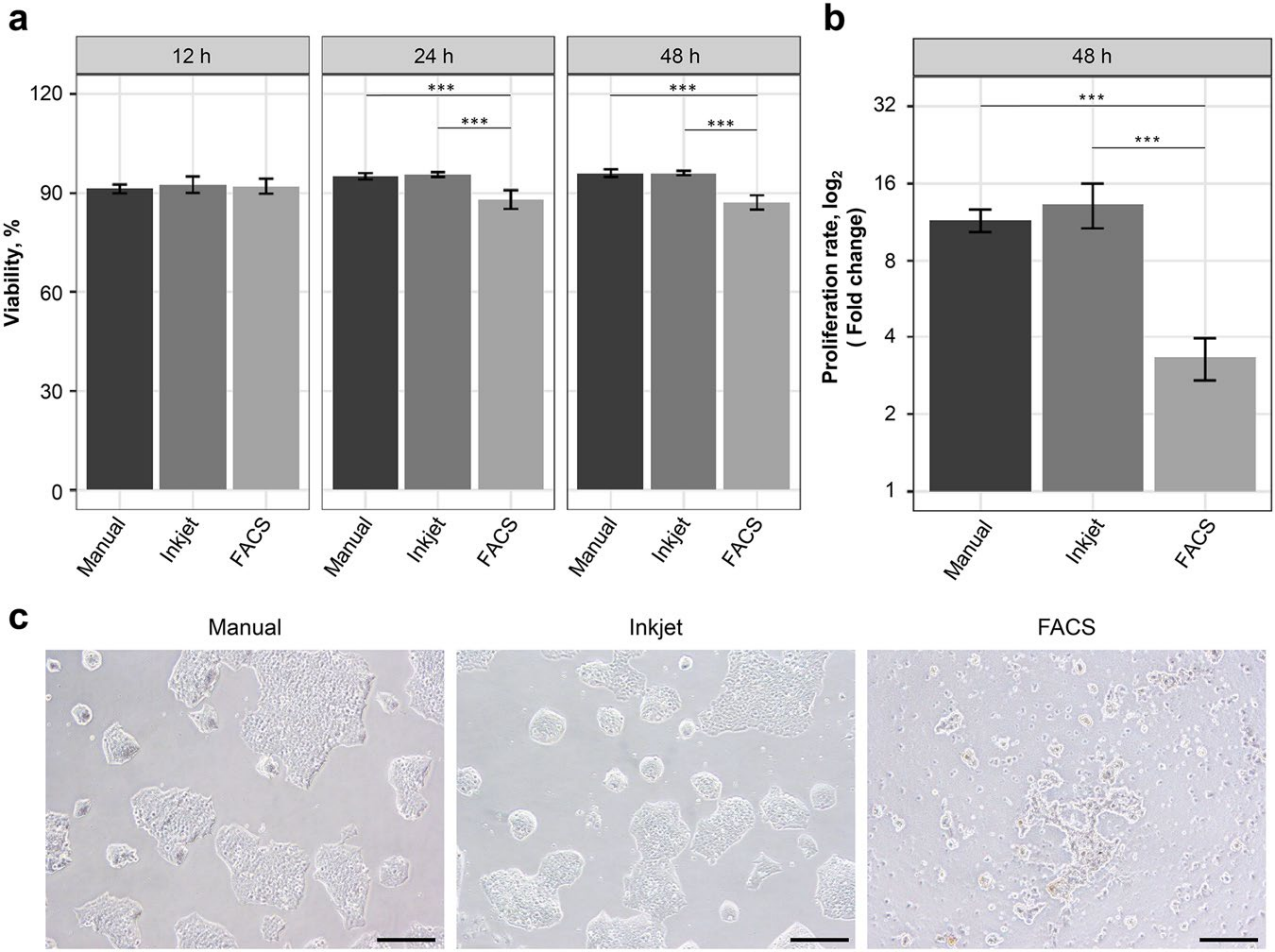
mESCs viability, proliferation and colony shapes. (**a**) Viability of mESCs cultured for 12, 24 and 48 h after dispensing using three methods. Blocks represent the viabilities of 12, 24 and 48-h culture cells from left to right, sequentially. Results are presented as mean ± SD. ***p.adjust < 0.001. (**b**) Proliferation rate at 48 h based on 12-h culture cells. Results are presented as mean ± SD. ***p.adjust < 0.001. (**c**) Microscopic images of mESCs colonies after 72-h culture. Scale bars are 200 μm.

Regarding cell dispensing using the droplet-based method, there are concerns over shear stress when the cells pass through a thin nozzle and inertia force at the time of landing on a liquid surface or substrate. We analysed the viability and proliferation of mESCs cultured for 12, 24 and 48 h after dispensing them by manual, inkjet and FACS methods. The cell viability of samples cultured for 12, 24 and 48 h are illustrated in Fig. 2a. We confirmed that the average viabilities of the inkjet-dispensed samples were maintained at over 90% for 48 h. In addition, there were no significant differences in viability between the manually dispensed samples and the inkjet-dispensed samples. For the FACS-dispensed samples, the viabilities decreased slightly at 24 and 48 h, and were 88.0 and 87.1%, respectively. The viability differences between FACS-dispensed samples and the two other methods were significant at 24 and 48 h (***Tukey’s HSD, *p*.adjust <0.001). The proliferation rates were calculated based on the viable cell numbers of 12-h samples (Fig. 2b). The 48-h manually dispensed and inkjet-dispensed samples proliferated about 12 times more compared to the 12-h samples, although the FACS-dispensed samples proliferated only three times more, and there were significant differences between the proliferation of the FACS-dispensed samples and the proliferation of the samples dispensed using the two other methods (***Tukey’s HSD, *p*.adjust <0.001).

The inertial force is proportional to the square of the droplet velocity. In the present evaluation, the droplet velocity of the inkjet bioprinter was 0.6 m/s, and the droplet velocity of FACS is generally 5–25 m/s;^15,22^ even if the droplet velocity is underestimated, FACS would have more inertia of approximately two orders of magnitude compared with our inkjet bioprinter. However, the impact of the inertia force is reduced by the droplet cushion because both inkjet and FACS have a droplet diameter >100 μm^23^. The result that the FACS sample viability at 12 h is similar to the viability of the manual samples is supported by the above cushion theory (Fig. 2a). For the shear stress during printing cells, the mean shear rate during droplet generation of the inkjet head was calculated as 1.1 × 10^4^ s^−1^ using velocity of the droplets and their radius^24^. In the same way, the share rate of FACS was calculated at 1 × 10^5^–5 × 10^5^ s^−1^. In the FACS system, dispensed cells might be exposed not only the shear stress from the nozzle, but also from a long tube of sample line. Therefore, the shear rate in FACS may be higher than that in inkjet bioprinter developed in our research. Regarding the effect of shear stress on cells, it has been reported that the cell growth rate^17^ is affected. Because there was no significant difference between the manual and inkjet method in terms of cell proliferation (Fig. 2b), it was indicated that our developed inkjet head exerts quite less shear stress on the cells.

To evaluate the impact of dispensing methods on colony formation, we observed the morphology of 72-h cultured samples using a microscope. As illustrated in Fig. 2c, the colony morphology of inkjet-dispensed and manually dispensed samples seemed to have normal shapes; however, the colonies of FACS-dispensed samples seemed to have abnormal small shapes. From the abnormal colony formation FACS results (Fig. 2c), to confirm the influence of sheath fluid during the culturing of mESCs, we dispensed mESCs manually and cultured them for 72 h using the solution prepared by adding 4 μl sheath fluid into 200 μl medium. This volume of 4 μl is nearly equivalent to 3,000 droplets of FACS dispensed using the nozzle of 100 μm diameter. The ratio of dead cells increased in the sample in which sheath fluid was added compared to the sample without sheath fluid (Supplementary Fig. S1). Although it was challenging to elucidate the mechanism of formation of abnormal shapes in the colonies in the FACS-dispensed samples because of the inability to isolate the influence of mechanical stress and sheath fluid, we believe that decreased viability after 24 h in the FACS-dispensed samples could have been the product of the mechanical stress and chemical stress associated with sheath fluid^14^.

### Conventional evaluations of the dispensed mESCs: maintenance of pluripotency

Generally, stem cells tend to differentiate in response to mechanical stress^25–27^. In contrast, shear stress may inhibit the induction of normal differentiation^28^. There are concerns about the effects of fluid shear stress when cells pass through the nozzle of inkjet heads and the inertial force during the landing of droplets, including that of cells on the media in the well plates^17,23,29^. Therefore, we evaluated the maintenance of pluripotency in post-dispensed mESCs using ALP assay, which can be used to confirm the undifferentiated state while maintaining cell and colony shapes, and qPCR to confirm differentiation marker genes.

**Figure 3:**
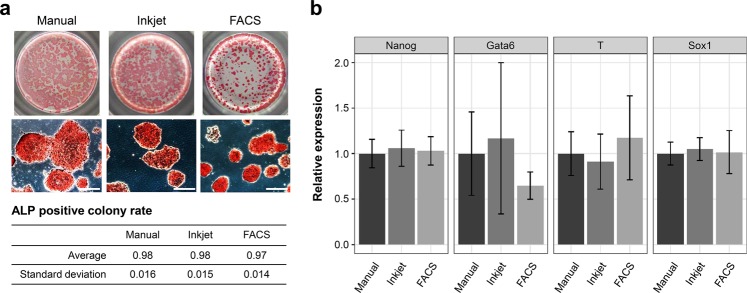
Assessment of the maintenance of pluripotency in dispensed mESCs. (**a**) Alkaline phosphatase activity of 72-h post-dispensed mESCs. Top images show the macroscale images of ALP-stained mESCs. Middle images show the microscopic images of ALP-stained mESCs. Scale bars are 200 μm. Bottom table shows the average and standard deviation of ALP-positive colony rates. (**b**) Relative expression of ES markers and markers of the three germ layers. log_2_ (−ΔΔ*C*_T_) values were normalised on the basis of the manual samples. Results are presented as mean ± SD.

mESCs dispensed by the three methods and cultured for 3 d in 96-well plates with differentiation suppressive medium were stained with ALP and observed macroscopically and microscopically (Fig. 3a). The undifferentiated state was maintained in the samples dispensed using all three methods. Similarly, there were no significant differences in ALP-positive colony formation rates, based on the numbers of ALP-positive/negative colonies, among the three cell-dispensing methods (Fig. 3a).

To confirm that the inkjet-dispensed mESCs maintained abilities to differentiate into all three germ layers, we induced the differentiation of the embryo bodies (EBs) of mESCs dispensed by the three methods and evaluated the differentiation markers using qPCR. Total RNA of the control cells that had not induced differentiation and of differentiated EBs were extracted and reverse transcribed into cDNA, and then the cDNA was quantified using a qPCR System. The expressions of gene markers were relatively quantified using the ΔΔ*C*_T_ method (Fig. 3b). We confirmed that compared with the differentiation markers of the control samples, those of the three germ layers (*Gata6* of endoderm marker, *T* of mesoderm marker and *Sox1* of ectoderm marker) were upregulated. To compare the effect of inkjet cell dispensing on mESCs, the ΔΔ*C*_T_ values were normalised using data from the manual samples. According to the results, there were no significant differences among all three methods, although *Gata6* as an endoderm marker for inkjet-dispensed sample fluctuated slightly (SD = 0.83). Based on the above results of pluripotency evaluations, we confirmed that, as with the manual method, the developed inkjet bioprinter did not affect the pluripotency of the mESCs.

### RNA-seq analysis for evaluating the effects of dispensing process on gene expression

Several studies have evaluated the effects of bioprinters on cells; however, very few studies have delved into cell integrity at the transcriptome level. Campbell *et al*. performed RNA-seq on MCF7 breast cancer cells (BCC) dispensed by the manual and thermal inkjet bioprinter^20^. In that report, the apoptotic rate of post-bioprinted MCF7 BCC at 24 h was 69%, and five upregulated differentially expressed genes related to ‘cellular response to stimulus’ of Gene Ontology biological processes were found in the bioprinted cells by RNA-seq analysis. These results indicate that heat shock effects should be considered in thermal inkjet bioprinters. We performed the RNA-seq to investigate the effect on cells specific to our inkjet bioprinter used piezoelectric-based membrane vibration system.

We conducted RNA-seq analyses of the control samples and dispensed samples and compared the variations in gene expressions. To confirm the time-course variation in gene expression based on the dispensing methods, we performed RNA-seq analyses on the cultured cell samples for 1, 2, 4, 6, 9, 12, 24, 48 and 72 h after dispensing. We assessed the quality of the sequence data and adopted three biological replicates for every time point and method.

**Figure 4:**
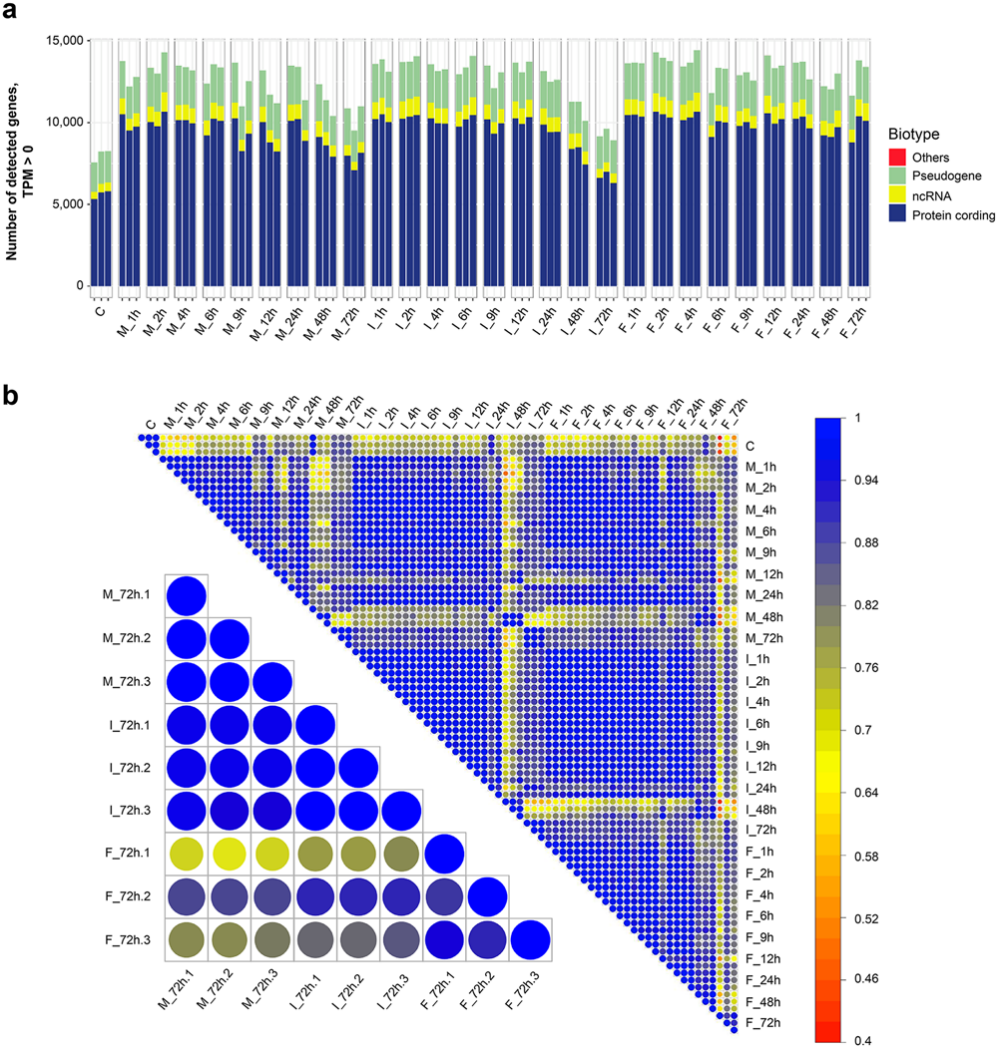
Number of genes detected before and after cell dispensing by RNA-seq, and the Pearson’s correlation coefficient for the time points and methods. (**a**) Number of detected genes and biotypes. From the left, control samples (C), manual method samples (M), inkjet method samples (I) and FACS method samples (F) are arranged in the order of time courses based on three biological replicates. Detected genes are categorised into biotypes of protein-coding genes, ncRNA, pseudogenes and others. (**b**) Correlation matrix for time points with three biological replicates represented using a colour scale ranging from 0.4 to 1. The circle sizes emphasise the magnitude of the correlation coefficients. Top-right side image shows the correlation matrix for the samples of time points and methods. Bottom-left side image shows the correlation matrix for the 72 h cultures.

Figure 4a presents a bar graph of the number of detected genes and biotypes. In the control samples, approximately 8,000 genes were detected and approximately 70% of the genes were protein-coding genes; moreover, 25% of the genes were ncRNA and 5% of the genes were pseudogenes. The number of genes detected in the 1-h sample post-dispensing markedly increased (over 10,000) in all methods and the number was remained increased state. Based on the number of genes detected using RNA-seq, gene expression in dispensed mESCs increased about 1.7-fold compared with the gene expression levels in the undispensed control samples (Fig. 4a), which implies that the passage process influences cells considerably because the manual method is almost equivalent to passage. Correlation analysis was performed to evaluate the variation in gene expression between every dispensing method and time point. The Pearson’s correlation coefficients among all the samples were calculated, and they had positive correlations (r = 0.419–0.996, *p* < 0.001) (Fig. 4b). On comparing the dispensed samples with the control samples, there were low correlations from 1 to 6 h samples for the manual and inkjet methods, and the correlations gradually increased up to 72 h. This means that the variation in gene expression immediately after dispensing due to the cellular dispensing process of inkjet and FACS was small and comparable to the manual method. In addition, when we focused on 72-h samples, there was a higher correlation between the manual and inkjet samples (r = 0.948–0.979) than between the manual and FACS samples (r = 0.687–0.880) (Fig. 4b, Supplementary Table S1). These results indicate that the transient stress of the developed inkjet bioprinter was at the same level as that of the manual method, and the state of the inkjet-dispensed cells at 72 h was very close to that of the conventional passage treated cells performed with a micropipette.

**Figure 5:**
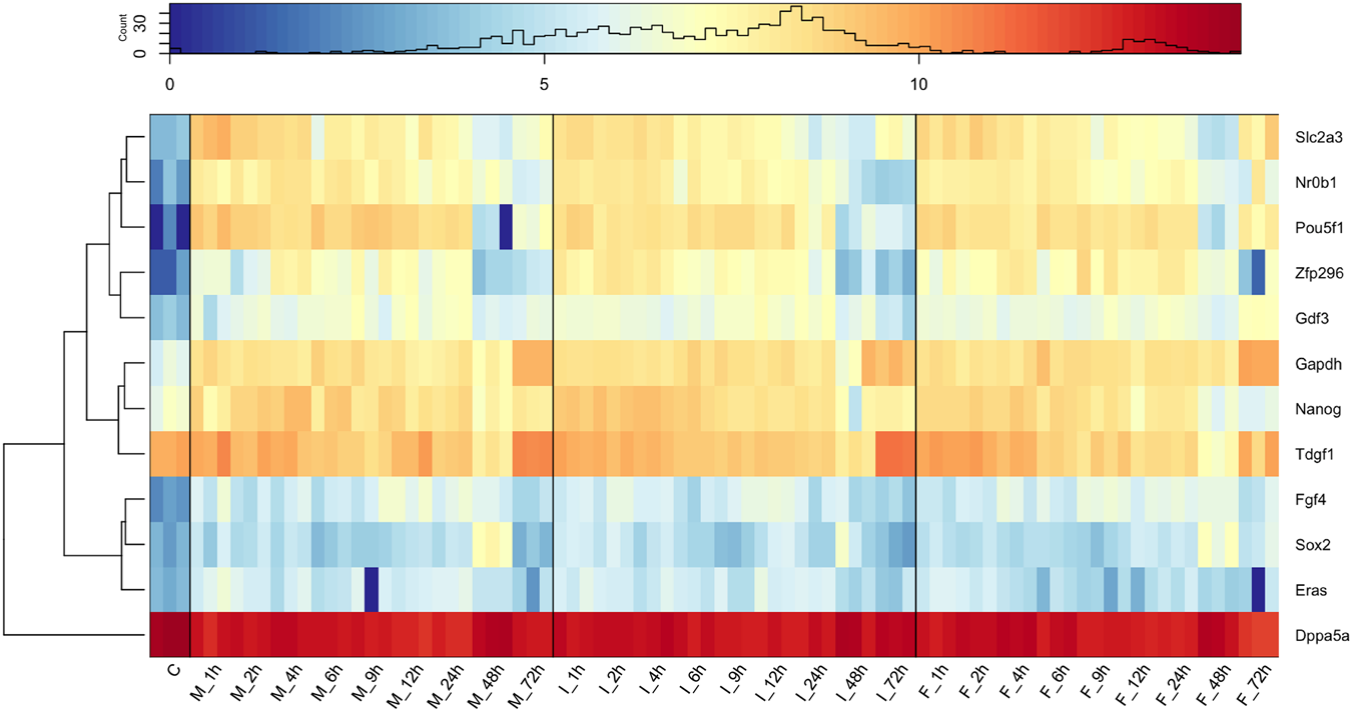
Heatmap of undifferentiated marker gene expressions. Data from the control (C) and post-dispensed samples: manual method (M), inkjet method (I) and FACS method (F) are mapped in the order of time courses with three biological replicates. Colour keys (blue/red) represent log_2_(TPM + 1). As a reference, *Gapdh* of housekeeping gene was also displayed. The histogram of TPM values is shown in the colour keys. TPM: transcriptome per million.

Whether stem cells dispensed using an inkjet bioprinter would maintain an undifferentiated state for an appropriate time and possess pluripotency to differentiate into the three germ layers are the important key points for evaluating the use of inkjet bioprinters in stem cell research. We evaluated the expression levels of embryonic stem cell (ESC) markers in the control undispensed and post-dispensed mESCs samples. Figure 5 shows a heatmap of the gene expression levels of these ESC markers. We observed a high expression of *Dppa5a* in all methods and at all time points. We confirmed that the gene expressions of ESC markers at almost all time points were not lower than control samples for all dispensing methods (Fig. 5). In addition to the number of ALP-stained mESC colonies (Fig. 3a), this RNA-seq result, which exhaustively analysed ESC markers, indicates that the inkjet-dispensed mESCs could maintain an undifferentiated state even if cultured for a period adequate for performing passaging. Furthermore, owing to the induction of differentiation into EBs, the markers of all three germ layers were upregulated from the undifferentiated mESCs and there were no significant differences (Fig. 3b). Consequently, the inkjet bioprinter does not affect mESC pluripotency with regard to the maintenance of the undifferentiated state and the capacity to differentiate into three germ layers.

**Figure 6:**
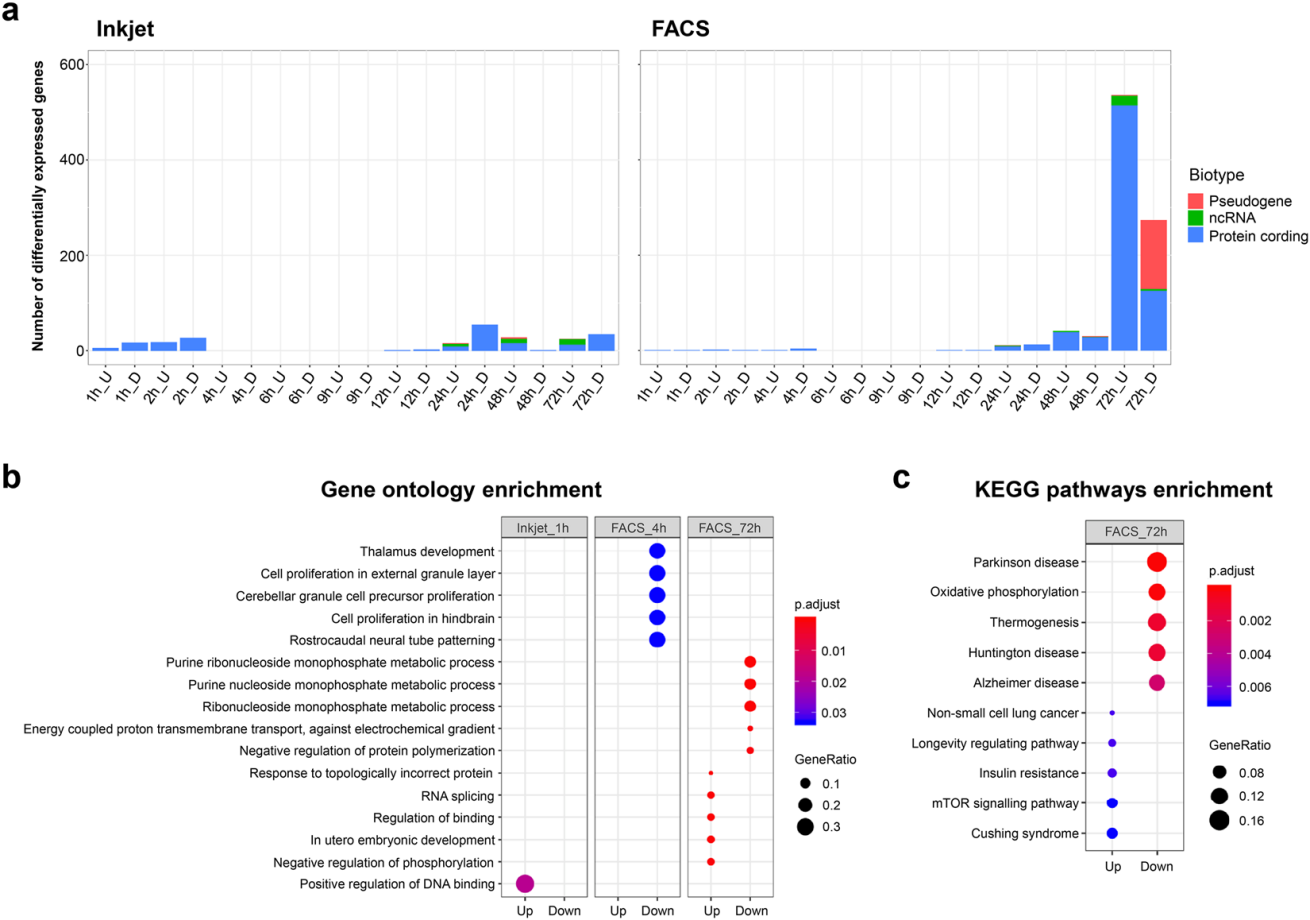
Differentially expressed genes (DEGs) obtained by comparing Inkjet and FACS methods with the manual method, and the enrichment analysis of DEGs. (**a**) Bar graph for the number of DEGs for comparing inkjet and FACS samples with manual samples. Left is inkjet DEGs and right is FACS DEGs. Colour represents the biotype of genes. (U) represents upregulated and (D) represents downregulated. (**b**) Dot plots of Gene Ontology enrichment results. (**c**) Dot plots of the KEGG pathway enrichment analysis results. GeneRatio are represented by the diameters of the circles, and *p*.adjust values are represented by colour.

Differentially expressed genes (DEGs) that significantly varied with time course were extracted by the likelihood ratio test using edgeR. When the DEGs varied with respect to the previous time point were extracted by the likelihood ratio test, there were over 3,000 DEGs in all methods at the period of 1 h after dispensed, and no remarkable variation from then (Supplementary Fig. S2). This variation in the number of DEGs were in agreement with the results of Fig. 4a, and also there was almost no variation of biotype until 24 h in all methods. In order to confirm the effect of inkjet dispensing, DEGs that significantly varied relative to the manual method were extracted by the likelihood ratio test. Figure 6a and Supplementary Table S2 show the number and biotypes of DEGs, which had significant differences compared to the manual samples. The number of DEGs after dispensing from 1 h to 6 h was not substantial. On the other hand, the 72-h FACS sample contained 810 DEGs. These results indicate that there were few differences between the cellular response to the inkjet method and its response to the manual method. The FACS method also had few differences to the manual method until 48 h; however, there appeared to be some difference in the cellular activity of mESCs after 72 h of culturing.

We performed enrichment analysis to confirm the type of cellular activity-related genes in the detected DEGs represented in Fig. 6a. Figure 6b presents the results of the Gene Ontology (GO) enrichment analysis, and Fig. 6c presents the KEGG pathway enrichment analysis. In the GO enrichment analysis, one term at 1 h was enriched for the inkjet method, and some terms at 4 h and 72 h were enriched for the FACS method. In the KEGG pathway enrichment analysis, some pathways were enriched only in FACS 72-h samples. The only enriched GO term in the inkjet samples was ‘positive regulation of DNA binding’. This term was enriched by the significant expression of *Pou4f2* and *Plaur* (*p*.adjust = 0.019). However, it is not readily discernible how the two genes are specifically linked to inkjet cell-dispensing effects. Therefore, there were no specific GO terms and pathways that were particularly associated with effects of inkjet dispensing.

At 4 h in the FACS samples, five brain-related terms such as ‘Thalamus development’, ‘cell proliferation in external granule layer’ and ‘rostrocaudal neural tube patterning’ were enriched in the downregulated genes in GO. However, these terms were enriched because *Gbx2* was significantly expressed (*p*.adjust = 0.033). *Gbx2* is known as a brain-related marker and an undifferentiated marker^30^. Therefore, it is presumed that these brain-related terms were enriched by significantly expressed *Gbx2* as an undifferentiated marker rather than a marker of brain-related functions.

In the FACS 72-h samples, there were five upregulated and five downregulated terms in both GO and KEGG pathway enrichment analyses. It seems that the upregulated genes were associated with cell growth (Fig. 6c) because ‘Non-small cell lung cancer’ was enriched by cell growth and tumour-related genes. In addition, ‘Longevity regulating pathways’, ‘Insulin resistance’ and ‘mTOR signalling pathway’ are pathways relating to the mTOR pathway. The mTOR pathway regulates cell growth and metabolism^31,32^. Conversely, downregulated DEGs seem to be related to ATP or mitochondrial activities (Fig. 6b,c) because the purine nucleoside metabolic process related terms related to ATP were enriched in GO. In addition, downregulated disease-associated pathways were enriched by mitochondrial respiratory chain complex-related genes such as *mt-Nd1*, *mt-Co1* and *Atp5g3*. According to the above enrichment analysis results, the 72-h FACS samples could have had cell growth activities that were not observed in the manual and inkjet samples. The reasons for cell growth-related gene expression in the 72-h samples of the FACS were not investigated in depth because it was not a major objective in the present study; however, it could be the effect of mechanical stress, which did not emerge immediately after dispensing, or the effect of culturing in a medium containing approximately 2% sheath fluid for 48 h.

Such differences in gene expression not being observed in the inkjet method compared with the FACS method is because of the following two merits: first, the inkjet head is designed to reduce mechanical stresses via a low droplet ejection velocity and lack of long narrow channels, and second, the inkjet head can dispense cells using only the medium. In other words, it does not require additional solutions such as sheath fluid or hydrogels.

In conclusion, a novel inkjet-based bioprinter that can print living cells without damaging them is evaluated in detail at the gene expression level. We demonstrated that the developed inkjet bioprinter is a cell-friendly dispensing method exhibiting no significant differences from the manual method based on aspects such as cell viability, pluripotency maintenance and gene expression. This conclusion is because of the mechanical stress-free design and chemical stress-free ability to dispense cells by using the medium as is. The developed inkjet bioprinter could facilitate researchers attempting to develop 3D organs for regenerative medicine or attempting to accelerate drug discovery via the efficient screening of candidate drugs since bioprinters often employ stress-sensitive cells such as stem cells and primary cells.

## Materials and Methods

### mESCs culture

mESCs 129/SVEV were purchased from Merck (Darmstadt, Germany) and cultured according to the manufacturer’s instructions. Cells were maintained in ESGRO Complete PLUS Clonal Grade medium (Merck) pre-coated with ESGRO Complete Gelatin (Merck). Cells were incubated in 5% CO_2_ at 37 °C. mESCs were dissociated using accutase (Merck) to allow them to be passaged routinely every 3 d.

### Dispensing mESCs using three methods

The cell suspensions were dispensed using three dispensing methods, including manual dispensing using a micropipette as the control, using an inkjet bioprinter and using FACS Aria™ III (BD Biosciences, San Diego, CA, USA), which is a high-speed dispensing system used extensively. The manual dispensing was performed used a single channel pipette. The inkjet head used in the study was developed and manufactured in house. We used the developed inkjet bioprinter under the following conditions: 100 Hz droplet frequency and 0.6 m/s droplet ejection velocity. The cell suspension was stored at 4 °C until just before to dispensing, and the cell suspension was dispensed in a short time after poured into the room temperature chamber of inkjet head. The FACS Aria III was used under the following conditions: a nozzle diameter of 100 μm, a flow rate setting of 1 equivalent to 10 μL/min, a sample holding temperature of 4 °C and the agitation function set to OFF. mESCs were dispensed into different well plates. All the mESCs dispensed into well plates were incubated in 5% CO_2_ at 37 °C.

### Viability and proliferation assay

mESCs were collected from dishes and centrifuged at 1,200 rpm for 1 min (KOKUSAN, Saitama, Japan), and then the cells were suspended in the mESC medium. After counting the cells, they were diluted to 2.0 × 10^6^ cells/mL for the manual and inkjet methods, and to 3.0 × 10^6^ cells/mL for the FACS method. The cells were dispensed using the three methods and plated on gelatin-coated 24-well plates at a concentration of 1.0 × 10^4^ cells/well, incubated in 5% CO_2_ at 37 °C, cultured for 12, 24 and 48 h, then Hoechst 33342 (Thermo Fisher Scientific, MA, USA) and Propidium Iodide (Sigma-Aldrich, Saint Louis, MO, USA) were added into the medium, incubated in 5% CO_2_ at 37 °C for 10 min, and washed using D-PBS (Thermo Fisher Scientific) once. As described in the mESC maintenance culture, mESCs were dissociated in 24-well plates and allowed to stand for 30 min at room temperature. Finally, the cells were counted under a BZ-X fluorescence microscope (KEYENCE, Osaka, Japan). All sample dispensing procedures using the three methods were performed in three biological replicates and four technical replicates. Proliferation rates were calculated using the living cell number divided by the counted cell number for viability analysis.

### Differentiation methods

The mESCs were collected and centrifuged at 1,200 rpm for 1 min (KOKUSAN), and then the cells were suspended in mESC medium. After counting the cells, they were diluted to 2.0 × 10^6^ cells/mL for the manual and inkjet-dispensing methods, and to 3.0 × 10^6^ cells/mL for the FACS dispensing method. Cells (2.0 × 10^3^) were dispensed using the three methods and plated on 10 wells of U-bottom 384-well plates (Sumitomo Bakelite, Tokyo, Japan) for every method to prepare EBs. The mESC media were changed every 2 d. After 5 d, the EBs were collected from every well and transferred to six-well plates, and the media were also changed with EmbryoMax DMEM (Merck). After 9 d, the EBs were collected for qPCR analyses.

### Relative quantification for pluripotency by qPCR

Total RNA of the control mESCs samples and the differentiated samples were extracted using an RNeasy Mini Kit (QIAGEN, Hilden, Germany) according to the manufacturer’s instructions. The concentrations of the extracted total RNA were measured using Qubit™ RNA HS Assay Kit (Thermo Fisher Scientific) and diluted using UltraPure™ distilled water (Thermo Fisher Scientific) to concentrations of 100 ng/μl. Subsequently, 100 ng of the total RNA samples were reverse transcribed into cDNAs using SuperScript® IV Reverse Transcriptase kits (Thermo Fisher Scientific) according to the manufacturer’s instructions. cDNAs were analysed using a QuantStudio™ 12 K Flex Real-Time PCR System (Thermo Fisher Scientific) with PrimeTime® Gene Expression Master Mix (Integrated DNA Technologies [IDT], IA, USA) and PrimeTime® qPCR Assays (IDT). The gene markers selected were *Nanog* as an embryonic stem cells marker, *Gata6* as an endoderm marker, *T* as a mesoderm marker, *Sox1* as an ectoderm marker and *Actb* and *Gapdh* as housekeeping gene markers. For a total volume of 10 μl in MicroAmp™ Optical 384-Well Reaction Plate with Barcode (Thermo Fisher Scientific), the reaction mixture was composed of 0.5 μl of *Actb* PrimeTime® qPCR Assays (20×) for internal control, 0.5 μl of PrimeTime® qPCR Assays (20×) for the other markers, 5 μl of PrimeTime® Gene Expression Master Mix (2×), 2 μl of UltraPure™ distilled water and 2 μl of template cDNAs. The reaction mixtures were heated at 50 °C for 2 min and 95 °C for 10 min, followed by 40 cycles of 95 °C for 15 s and 60 °C for 1 min. For relative quantification, the Δ*C*_T_ values were calculated by comparing *Actb C*_T_ values with *C*_T_ values of the other marker genes, and then the 2^−(ΔΔ*C*T)^ values were calculated comparing the induction-differentiated samples with the control mESCs samples. All samples dispensed by three methods had three biological replicates and four technical replicates.

### Alkaline phosphatase staining

mESCs were cultured for 3 d and ALP was detected using Vector Red alkaline phosphatase substrate (Vector Laboratories, Burlingame, CA, USA). Stain solution was prepared according to the manufacturer’s instructions, and then washed with 200-mM Tris-HCl, pH 8.2–8.5 buffer (Wako, JAPAN) and then the stain solution added to the wells. The samples were incubated with the substrate working solution for 30 min in the dark at room temperature. Finally, the cells were washed with Tris-HCl buffer for 5 min and rinsed in water.

### RNA-sequencing

We dispensed 3.0 × 10^3^ mESCs into 96-well plates using the three dispensing methods. Before dispensing using each method, 500 cells were sampled per dish as the control, and after culturing for 1, 2, 4, 6, 9, 12, 24, 48 and 72 h, 500 cells were sampled per well. The cDNA libraries were constructed according to the Smart-seq. 2 method^33^. In the sequencing procedure, an Illumina Miseq system (Illumina, San Diego, CA, USA) was with Miseq Reagent Kit v3 (150 Cycles) (Illumina). All samples were had four biological replicates.

### Sequencing data analysis

RNA-seq reads were adaptor trimmed with flexbar^34^ v 3.3.0 before mapping to the mouse reference genome Ensembl Mus musculus (Release 94) with hisat2^35^ v2.1.0. StringTie^36^ v 1.3.4 d was used to estimate gene expression levels. Three replicate data were adopted after quality checks on sequence data. The numbers of detected genes were counted for genes of transcriptome per million (TPM) > 0. Detected genes were categorised by biotypes. For the correlation analysis with the Pearson’s correlation coefficient, protein-coding genes with TPM > 1 were used. For the evaluation of a maintenance undifferentiated state, the ESC markers were selected according to advance research by Takahashi *et al*. (2016)^37^. Differential gene expression analyses were performed using edgeR^38^, and the following threshold parameters were adopted: TPM > 1, FDR < 0.05, *p* < 0.05 and |log_2_FC|> 2. For the DEGs, we conducted enrichment analyses for Gene Ontology biological processes and the KEGG pathway using clusterProfiler^39^ under the following conditions: pvalueCutoff = 0.05, qvalueCutoff = 0.01. In order to calculate *p*.adjust values for multiple hypothesis testing, the Benjamini and Hochberg method was selected. The GeneRatio is the number of genes in input gene list associated with the given GO term. In addition, simplify function was used to cut down redundant words for the GO enrichment at a threshold of 0.4.

### Statistical analysis

Viability, proliferation rate, qPCR and ALP assay data are presented as means ± standard deviation. Significant differences among the three dispensing methods were evaluated using Tukey’s HSD test. Probability values of *p* < 0.05, 0.01 and 0.001 were considered statistically significant.

### Accession number

The sequence data have been deposited in DNA Data Bank of Japan (DDBJ) under the accession number DRA009710.

## Supplementary information

Supplementary information.

## Data Availability

The datasets analysed during the current study are available from the corresponding authors on reasonable request.
