# Supplementary material for: Evaluation of the effects of cell-dispensing using an inkjet-based bioprinter on cell integrity by RNA-seq analysis
Source: Sci Rep. 2020 Apr 28;10:7158. doi: 10.1038/s41598-020-64193-z (PMC7189371; doi:10.1038/s41598-020-64193-z)
Supplement: Supplementary file 1 — Supplementary information. [file 41598_2020_64193_MOESM1_ESM.pdf]

Supplementary Information: Evaluation of the effects of cell-dispensing using an inkjet-based bioprinter on cell integrity by RNA-seq analysis

AUTHORS AND AFFILIATONS

Masayuki Yumoto[1,2], Natsuko Hemmi[2], Naoki Sato[2], Yudai Kawashima[2], Koji Arikawa[5], Keigo Ide[1,3], Masahito Hosokawa[4,5], Manabu Seo[2] & Haruko Takeyama[1,3,4,5,*]

[1]Department of Life Science and Medical Bioscience, Waseda University, 2-2 Wakamatsu-cho, Shinjuku-ku, Tokyo, 162-8480, Japan

[2]Biomedical Business Center, Healthcare Business Group, Ricoh Company, Ltd., 3-25-22 Tonomachi LIC 322, Kawasaki, Kanagawa 210-0821, Japan

[3]Computational Bio Big-Data Open Innovation Laboratory, AIST-Waseda University, 3-4-1 Okubo, Shinjuku, Tokyo, 169-0072, Japan

[4]Institute for Advanced Research of Biosystem Dynamics, Waseda Research Institute for Science and Engineering, Waseda University,
2-2 Wakamatsu-cho, Shinjuku-ku, Tokyo 162-8480, Japan

[5]Research Organization for Nano and Life Innovation, Waseda University, 513 Waseda-tsurumaki-cho, Shinjuku-ku, Tokyo 162-0041, Japan

***Corresponding author:**
Haruko Takeyama,
Department of Life Science and Medical Bioscience, Waseda University
2-2 Wakamatsu-cho, Shinjuku-ku, Tokyo 162-8480, Japan
Telephone No.: +81-3-5369-7326
Fax No.: +81-3-5369-7326
E-mail address: haruko-takeyama@waseda.jp

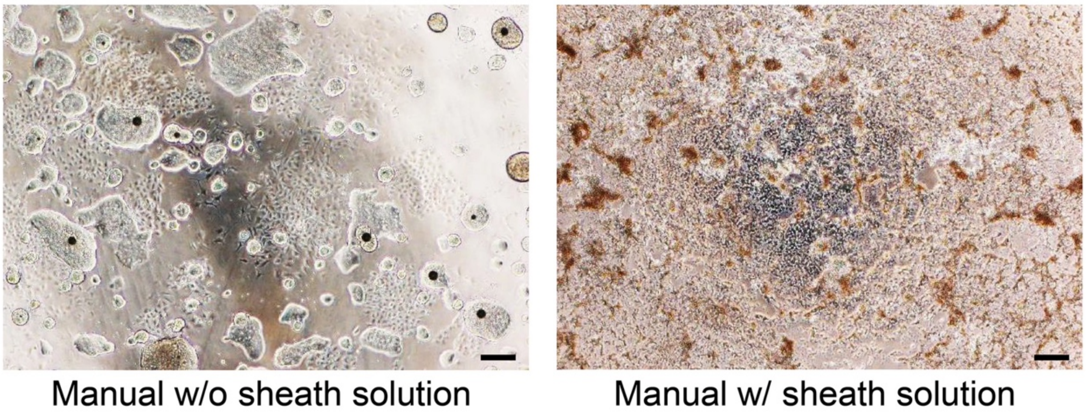

Supplementary Figure S1. Evaluation of sheath fluid during culturing of mESCs. Left image displays the colonies cultured for 72 h and dispensed using the manual method in 200 µl medium. Right image displays the colonies cultured for 72 h and dispensed using the manual method in medium similar to the medium used in the left image with sheath fluid (4 µl) added that was equivalent to 3,000 droplets of FACS dispensing. Scale bars represent 200 µm.

Supplementary Table S1. Correlation matrix for 72-h samples. 'M' means manual, 'I' means inkjet and 'F' means FACS. The number at the end represents replicate.

**Correlation**	**M_72h.1**	**M_72h.2**	**M_72h.3**	**I_72h.1**	**I_72h.2**	**I_72h.3**	**F_72h.1**	**F_72h.2**	**F_72h.3**
**M_72h.1**	1	0.995	0.995	0.973	0.979	0.962	0.703	0.88	0.795
**M_72h.2**	0.995	1	0.996	0.962	0.969	0.948	0.687	0.868	0.783
**M_72h.3**	0.995	0.996	1	0.963	0.973	0.952	0.712	0.878	0.803
**I_72h.1**	0.973	0.962	0.963	1	0.995	0.995	0.769	0.901	0.828
**I_72h.2**	0.979	0.969	0.973	0.995	1	0.991	0.771	0.902	0.836
**I_72h.3**	0.962	0.948	0.952	0.995	0.991	1	0.785	0.902	0.841
**F_72h.1**	0.703	0.687	0.712	0.769	0.771	0.785	1	0.886	0.948
**F_72h.2**	0.88	0.868	0.878	0.901	0.902	0.902	0.886	1	0.916
**F_72h.3**	0.795	0.783	0.803	0.828	0.836	0.841	0.948	0.916	1

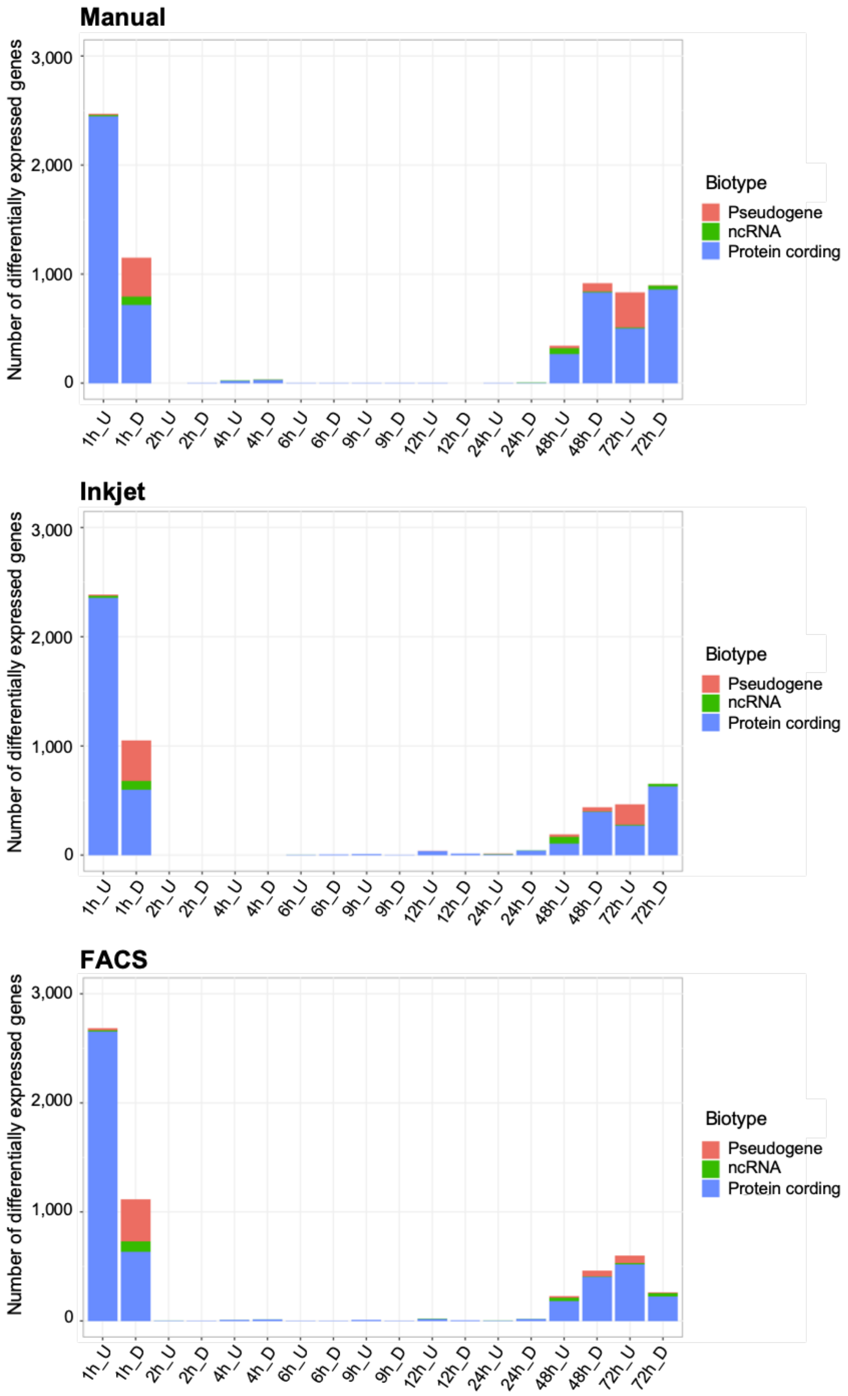

Supplementary Figure S2. Differentially expressed gene (DEG) obtained by the likelihood ratio test compared to the previous time point. Bar graph for the number of DEGs. Top is manual DEGs, middle is inkjet DEGs and bottom is FACS DEGs. Colour represents the biotype of genes. (U) represents upregulated and (D) represents downregulated.

Supplementary Table S2. Differentially expressed genes that significantly varied relative to the manual method.

Comparing Method	Culturing time	Upregulated genes				Downregulated genes			
		Protein cording	ncRNA	pseudogene	Total	Protein cording	ncRNA	pseudogene	Total
Manual versus inkjet	**1 h**	6	0	0	6	17	0	0	17
	**2 h**	18	0	0	18	27	0	0	27
	**4 h**	1	0	0	1	0	0	0	0
	**6 h**	0	0	0	0	0	0	0	0
	**9 h**	0	0	0	0	0	0	0	0
	**12 h**	1	0	0	1	3	0	0	3
	**24 h**	9	12	8	29	57	0	0	57
	**48 h**	74	12	80	166	12	2	1	15
	**72 h**	13	11	1	25	35	0	0	35
Manual versus FACS	**1 h**	1	0	0	1	1	0	0	1
	**2 h**	2	0	0	2	1	0	0	1
	**4 h**	1	0	0	1	4	0	0	4
	**6 h**	0	0	0	0	0	0	0	0
	**9 h**	0	0	0	0	0	0	0	0
	**12 h**	1	0	0	1	1	0	0	1
	**24 h**	9	1	1	11	13	0	0	13
	**48 h**	39	2	0	41	29	1	1	31
	**72 h**	512	21	1	534	125	4	146	275
